# Surveillance study of the prevalence, species distribution, antifungal susceptibility, risk factors and mortality of invasive candidiasis in a tertiary teaching hospital in Southwest China

**DOI:** 10.1186/s12879-019-4588-9

**Published:** 2019-11-07

**Authors:** Zhang-rui Zeng, Gang Tian, Yin-huan Ding, Kui Yang, Jin-bo Liu, Jian Deng

**Affiliations:** Department of Laboratory Medical, Affiliated Hospital of Southwest Medical University, 25 Taiping street, Luzhou, 646000 People’s Republic of China

**Keywords:** Invasive candidiasis, Epidemiology, Antifungal susceptibility, Mortality, Risk factors

## Abstract

**Background:**

Invasive candidiasis (IC) is the most common invasive fungal infection. The epidemiology of IC in hospitalized patients has been widely investigated in many metropolitan cities; however, little information from medium and small cities is known.

**Methods:**

A 5-year retrospective study was carried out to analyze the prevalence, species distribution, antifungal susceptibility, risk factors and mortality of inpatients with invasive *Candida* infection in a regional tertiary teaching hospital in Southwest China.

**Results:**

A total of 243 inpatients with invasive *Candida* infection during the five-year study period were identified, with a mean annual incidence of 0.41 cases per 1000 admissions and a 30-day mortality rate of 12.3%. The species distributions of *Candida albicans*, *Candida glabrata*, *Candida tropicalis*, *Candida krusei*, *Candida parapsilosis* and other *Candida* species was 45.3, 30.0, 15.2, 4.9, 2.1 and 2.5%, respectively. The total resistance rates of fluconazole (FCA), itraconazole (ITR) and voriconazole (VRC) were 18.6, 23.1 and 18.5%, respectively. Respiratory dysfunction, pulmonary infection, cardiovascular disease, chronic/acute renal failure, mechanical ventilation, abdominal surgery, intensive care in adults, septic shock and IC due to *C. albicans* were associated with 30-day mortality (*P < 0.05*) according to the univariate analyses. Respiratory dysfunction [*odds ratio* (OR), 9.80; 95% confidence interval (CI), 3.24–29.63; *P* < 0.001] and IC due to *C. albicans* (OR, 3.35; 95% CI, 1.13–9.92; *P* = 0.029) were the independent predictors of 30-day mortality.

**Conclusions:**

This report shows that the incidence and mortality rates are lower and that the resistance rates to azoles are higher in medium and small cities than in large cities and that the species distributions and risk factors in medium and small cities are different from those in large cities in China. It is necessary to conduct epidemiological surveillance in medium and small cities to provide reference data for the surveillance of inpatients with IC infections.

## Background

Invasive candidiasis (IC) is the most common fungal disease among hospitalized patients worldwide. According to conservative estimates, IC affects more than 250,000 people worldwide every year and is the cause of more than 50,000 deaths [1]. IC is widely reported in critically ill patients in the intensive care unit (ICU) [2]. The risk factors for IC included granulocytopenia, stem cell transplant, organ transplants, broad-spectrum antimicrobial agents, central venous catheterization, total parenteral nutrition, length of stay in the ICU, surgery, advanced life support, and aggressive chemotherapy [2]. With the increase of in related research, there have been reports showing that older age (over 65 years) [3], diabetes mellitus and chronic renal failure [4] are identified as risk factors for patients with IC

IC is caused by *Candida* species and comprises both candidemia and deep-seated tissue candidiasis. Candidemia is the most frequent form of IC. More than 15 *Candida* species can cause human candidemia [1, 2]. Deep-seated candidiasis is caused by either hematogenous dissemination or the direct inoculation of *Candida* species to a sterile site, such as the peritoneal cavity. Globally, *Candida albicans* is the primary cause of candidemia and one of the most common species in many countries, including Japan (39.5%) [5], Italy (61.2%) [6], Russia (43.2%) [7], Saudi Arabia (38.3%) [8] and Mexico (40%) [9], among others. However, *Candida parapsilosis* (17.8%) is the most common cause of IC in Pakistan [10]. The global incidence of IC varies from 0.3 to 5 per 1000 admissions according to hospital-based studies [11]. The mortality rate of IC in immunocompromised and other critically ill patients is between 35 and 80% [12]. The cost of IC treatment reached US$ 17,000 per patient in China, which was significantly higher than that for patients without IC (US$ 8500; *P* = 0.001) [13].

In China, the epidemiology of IC varies widely among different areas [14]. *C. albicans* (44.9%) is the most common strain isolated from IC patients in China, particularly in metropolitan cities [15]. Non-*C. albicans* species (59.9%) also play an important role in IC [16]. Data regarding epidemiologic trends and patient susceptibility to invasive fungal infections has mainly been studied in metropolitan cities, and little information from medium and small cities is known. Therefore, in the present study, we performed a five-year retrospective study to evaluate the epidemiology, antifungal susceptibility, risk factors and mortality of IC in patients in a tertiary teaching hospital in Southwest China.

## Methods

### Patient data collection

We conducted a retrospective observational study of electronic laboratory records. The fungal specimen data were collected from inpatients who were aged > 16 years with IC in the Affiliated Hospital of Southwest Medical University (Luzhou, China), which is a 3200-bed tertiary care teaching hospital with 43 wards and approximately 120,000 annual admissions, from January 2013 to December 2017. The diagnostic criteria of IC were based on the guidelines for the diagnosis and treatment and the Chinese expert consensus statement issued by relevant societies and organizations of the Chinese Medical Association [17–19]; these criteria were also in accordance with the revised definitions of invasive fungal disease (IFD) from the European Organization for the Research and Treatment of Cancer/Mycoses Study Group (EORTC/MSG) consensus group [20] and Infectious Diseases Society of America (IDSA) Guidelines for the Diagnosis and Management of Intravascular Catheter-Related Bloodstream Infection [21]. For each patient, only the first episode was included in our analysis. Patient cultures with two or more fungal species were excluded from the analysis, and all data were collected from electronic medical records. The following data were retrospectively collected from all the patients: demographic characteristics, underlying comorbidities, specific fungal pathogens and species, susceptibility to antifungal agents and survival. The following risk factors associated with IC were also collected: systemic corticosteroid treatment (a dose equivalent to prednisone 10 mg/d for at least 14 days), neutropenia (absolute neutrophil count < 500 cells/μl), abdominal surgery, indwelling central vascular catheter, mechanical ventilation, ICU hospitalization, concomitant bacterial infections, chemotherapy, total parenteral nutrition, septic shock, hemodialysis, broad-spectrum antibiotic therapy and treatment with antifungal agents. Early mortality was defined as death within 7 days, and late mortality was defined as death between 7 and 30 days. The study protocol was approved by the ethics committee of the hospital (Project No. K2016004). The need for informed consent was waived by the Clinical Research Ethics Committee.

### Microorganism identification and antifungal susceptibility

According to the manufacturer’s instructions, blood and sterile body fluid (including ascitic fluid, pleural fluid, cerebrospinal fluid and drainage fluid) were inoculated into both aerobic and anaerobic BacT/AlerT 3D vials (Bruker Diagnostics Inc., USA). The central venous catheter tips and sterile tissues were inoculated onto blood agar media (Columbia) and chocolate blood agar plates (+vancomycin) (Thermo Fisher Biochemical Product Co., Ltd., Beijing, China), respectively. All positive cultures were manually sampled and inoculated onto CHROMagar *Candida* medium (CHROMagar Company, France) to ensure viability and purity. The identification of all species was confirmed by a MicroScan WalkAway 96 Plus System (Siemens, Germany) and Microflex LT (Bruker Diagnostics Inc., USA) matrix-assisted laser-desorption/ionization time-of-flight (MALDI-TOF) mass spectroscopy (MS) system.

Antifungal susceptibility tests for fluconazole (FCA), itraconazole (ITR), amphotericin B (AMB), voriconazole (VRC) and flucytosine (5-FC), were performed for all *Candida* strain isolates by using an ATB FUNGUS 3 kit (bioMérieux, France). The minimal inhibitory concentrations (MICs) of the antifungal agents were judged by visual reading in our laboratory according to the manufacturer’s instructions. The quality control strains were *C. parapsilosis* ATCC 22019 and *C. krusei* ATCC 6258. The results were interpreted using the Clinical and Laboratory Standards Institute M27-A3 microbroth dilution method.

### Statistical analyses

The data were analyzed using SPSS software version 22 for Windows (SPSS, Chicago, IL, USA). The categorical data were compared using chi-square or Fisher’s exact tests. The continuous data were analyzed using Student’s *t*-test or Mann-Whitney U test. Statistical significance was determined using two-tailed tests, and *P* < 0.05 was considered statistically significant. Multivariable logistic regression analysis was performed to identify independent predictors of IC and 30-day hospital mortality. Biologically plausible variables with a value of *P* < 0.1 according to the univariate analyses were included in the multiple logistic regression model. For the independent predictors of 30-day hospital mortality, the following variables were entered into the model: respiratory dysfunction, pulmonary infection, cardiovascular disease, neurological diseases, chronic/acute liver disease, chronic/acute renal failure, mechanical ventilation, total parenteral nutrition, abdominal surgery, intensive care in adults, concomitant bacterial infections, septic shock, and *C. albicans*.

## Results

**Fig. 1:**
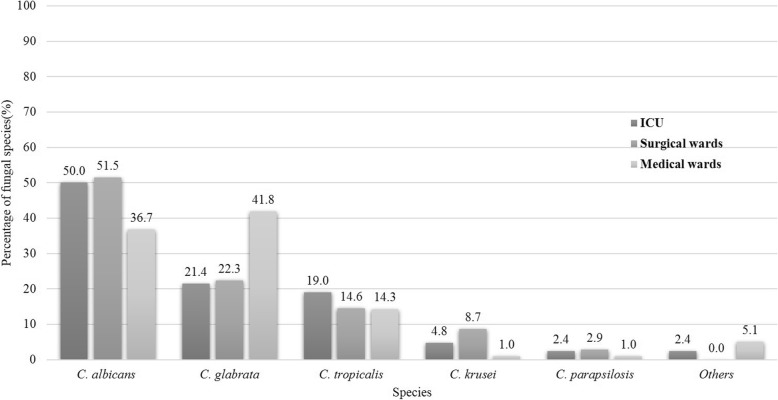
Distribution of the fungal species according to different wards. Others: *C. guilliermondii* (3), *C*. *haemulonii* (2) and *C*. *pseudotropicalis* (1)

A total of 243 distinct IC episodes were identified during our study period. The mean annual incidence of IC was 0.41/1000 admissions. A total of 209,611 cultures were also collected in our hospital; the positive rate of the fungal cultures was 4.3% (8963), 2.7% (243) of which corresponded to IC episodes from 21 wards. The culture-positive specimen came from the blood (116, 47.7%), sterile body fluid (98, 40.3%), central venous catheter tips (17, 7.0%) and sterile tissues (12, 4.9%). We classified all the wards into ICUs (42,17.3%), medical wards (103,40.3%) and surgical wards (98,42.4%). *C. albicans* was the predominant species in the ICU and surgical wards (50.0 and 51.5%, respectively), whereas *C. glabrata* was the predominant species (41.8%) in the medical wards (Fig. 1).

The cohort characteristics are described in Table 1; among the included patients (median age, 60 years), 57.2% were males. The percentages of the three most common isolated *Candida* species were as follows: *C. albicans* (45.3%), *C. glabrata* (30.0%) and *C. tropicalis* (15.2%) (Table 1). In patients in the age range of 49–65 years, *C. glabrata* was the predominant species (41.6%), but in patients in the age ranges of 16–49 and > 65, *C. albicans* was the main species (45.7 and 60.7%, respectively). The causative organism varied according to the age of the patients and their underlying diseases. With increasing age, the percentage of *C. glabrata* was reduced from 41.6 to 16.9%, and *C. albicans* and *C. tropicalis* significantly increased from 28.6 to 60.7% and from 11.4 to 15.7%, respectively. Non-*C. albicans* species were isolated with the highest frequency from patients with HIV/AIDS (84.6%); *C. glabrata* was the predominant species (76.9%). The incidence of IC increased from 0.16 episodes/1000 admissions in 2013 to 0.66 episodes in 2017, and the change in incidence over time was statistically significant (*P* < 0.05). A significant increase in the incidence (episodes/1000 admissions) of *C. albicans* (from 0.05 to 0.25), *C. glabrata* (from 0.06 to 0.23), *C. tropicalis* (from 0.03 to 0.12) and *C. parapsilosis* (from 0 to 0.03) was observed. The demographic and clinical characteristics of the patients are summarized in Table 1. Most of the patients had one or more comorbidities. The most common underlying comorbidities documented prior to IC were diabetes mellitus (38.3%), chronic/acute renal failure (37.0%), pulmonary infection (33.3%), cardiovascular disease (32.1%) and gastrointestinal pathology (25.9%). Moreover, the most common underlying conditions documented prior to IC were prior exposure to broad-spectrum antibiotics (88.5%), total parenteral nutrition (49.0%), central venous catheterization (48.6%), treatment with antifungal agents (37.6%) and mechanical ventilation (33.7%).

**Table 1:** Patient characteristics and incidence (episode/1000 admission)

	*Candida* species						
	Total	*C. albicans*	*C. glabrata*	*C. tropicalis*	*C. krusei*	*C. parapsilosis*	Others^h^
	(*n* = 243) 100.0%	(*n* = 110) 45.3%	(*n* = 73) 30.0%	(*n* = 37) 15.2%	(*n* = 12) 4.9%	(*n* = 5) 2.1%	(*n* = 6) 2.5%
Patient characteristics							
Age (years)							
16–49	70 (28.8)	32 (45.7)	23 (32.8)	8 (11.4)	3 (4.3)	2 (2.9)	2 (2.9)
50–65	84 (34.6)	24 (28.6)	35 (41.6)	15 (17.9)	6 (7.1)	2 (2.4)	2 (2.4)
> 65	89 (36.6)	54 (60.7)	15 (16.9)	14 (15.7)	3 (3.4)	1 (1.1)	2 (2.2)
Gender							
Male	139 (57.2)	60 (43.2)	41 (29.5)	28 (20.1)	4 (2.9)	4 (2.9)	2 (1.4)
Female	10 4 (42.8)	50 (48.1)	32 (30.8)	9 (8.7)	8 (7.7)	1 (0.9)	4 (3.8)
Underlying comorbidities (*n*, %)							
Gastrointestinal perforation	48 (19.6)	24 (50.0)	10 (20.8)	8 (16.7)	5 (10.4)	0 (0)	1 (2.1)
Respiratory dysfunction^a^	51 (21.0)	25 (49.0)	16 (31.4)	9 (17.6)	0 (0)	0 (0)	1 (2.0)
Pulmonary infection	81 (33.3)	43 (53.1)	20 (24.7)	14 (17.3)	1 (1.2)	1 (1.2)	2 (2.5)
Cardiovascular disease	78 (32.1)	41 (52.6)	20 (25.6)	10 (12.8)	5 (6.4)	0 (0)	2 (2.6)
Neurological diseases	58 (23.9)	35 (60.4)	10 (17.2)	9 (15.5)	2 (3.5)	1 (1.7)	1 (1.7)
Gastrointestinal pathology^b^	63 (25.9)	32 (50.8)	18 (28.6)	7 (11.1)	5 (7.9)	1 (1.6)	0 (0)
Chronic/acute liver disease	45 (18.5)	22 (48.9)	9 (0.2)	10 (22.2)	3 (6.7)	1 (2.2)	0 (0)
Chronic/acute renal failure^c^	90 (37.0)	48 (53.3)	23 (25.6)	15 (16.7)	2 (2.2)	0 (0)	2 (2.2)
Solid tumour	22 (9.1)	11 (50.0)	2 (9.1)	7 (31.8)	2 (9.1)	0 (0)	0 (0)
Haematological malignancy	6 (2.5)	3 (50.0)	1 (16.7)	1 (16.7)	0 (0)	0 (0)	1 (16.6)
Severe autoimmune diseases	11 (4.5)	7 (63.6)	0 (0)	3 (27.3)	1 (9.1)	0 (0)	0 (0)
Diabetes mellitus	93 (38.3)	57 (61.3)	19 (20.4)	11 (11.8)	6 (6.5)	0 (0)	0 (0)
Burns	6 (2.5)	2 (33.3)	1 (16.7)	1 (16.7)	0 (0)	2 (33.3)	0 (0)
HIV/AIDS	13 (5.3)	2 (15.4)	10 (76.9)	0 (0)	0 (0)	0 (0)	1 (7.7)
Severe trauma	15 (6.2)	6 (40.0)	3 (20.0)	4 (26.6)	1 (6.7)	1 (6.7)	0 (0)
Risk factors (*n*, %)							
Presence of CVC^d^	118 (48.6)	59 (50.0)	23 (19.5)	24 (20.3)	8 (6.8)	2 (1.7)	2 (1.7)
Mechanical ventilation	82 (33.7)	41 (50.0)	19 (23.2)	15 (18.3)	5 (6.1)	1 (1.2)	1 (1.2)
Receipt of corticosteroids^e^	70 (28.8)	43 (61.4)	12 (17.1)	14 (20.0)	1 (1.4)	0 (0)	0 (0)
Total parenteral nutrition	119 (49.0)	62 (52.1)	29 (24.4)	19 (16.0)	7 (5.9)	1 (0.8)	1 (0.8)
Chemotherapy	11 (4.5)	4 (36.4)	1 (9.1)	5 (45.4)	1 (9.1)	0 (0)	0 (0)
Abdominal surgery^f^	69 (28.4)	26 (37.7)	27 (39.1)	10 (14.5)	4 (5.8)	0 (0)	2 (2.9)
Intensive care in adults	42 (17.3)	21 (50.0)	9 (21.4)	8 (19.0)	2 (4.8)	1 (2.4)	1 (2.4)
Neutropenia^g^	7 (2.9)	4 (57.1)	0 (0)	1 (14.3)	2 (28.6)	0 (0)	0 (0)
Concomitant bacterial infections	81 (33.3)	46 (56.8)	13 (16.0)	18 (22.3)	2 (2.5)	1 (1.2)	1 (1.2)
Septic shock	46 (18.9)	16 (34.8)	19 (41.4)	7 (15.2)	2 (4.3)	0 (0)	2 (4.3)
Dialysis	25 (10.3)	9 (36.0)	13 (52.0)	2 (8.0)	0 (0)	0 (0)	1 (4.0)
Previous antibiotics therapy	215 (88.5)	103 (47.9)	60 (27.9)	35 (16.3)	12 (5.6)	1 (0.5)	4 (1.8)
Treatment with antifungal agents	83 (34.2)	44 (53.0)	20 (24.1)	14 (16.9)	3 (3.6)	0 (0)	2 (2.4)
Incidence (episodes/1000 admissions)							
2013	0.16	0.05	0.06	0.03	0.01	0	0.01
2014	0.31	0.16	0.07	0.06	0.01	0	0.01
2015	0.43	0.18	0.13	0.06	0.04	0.02	0
2016	0.52	0.28	0.13	0.05	0.03	0	0.03
2017	0.66	0.25	0.23	0.12	0.02	0.03	0.01

^a^Includes the following diseases: chronic obstructive pulmonary disease and acute respiratory distress syndrome

^b^Includes the following diseases: cholecystitis, pancreatitis, and peritonitis

^c^Chronic/Acute renal failure is the permanent or sudden and often temporary loss of kidney function with N waste retention and hypourocrinia

^d^*CVC* central venous catheter

^e^a dose equivalent to the prednisone dosage of 0.3 mg/kg/day for at least 14 days

^f^including: gastrointestinal perforations, severe acute pancreatitis and complex ventral hernia

^g^Neutropenia is the absolute neutrophil count, that is, < 500 cells/μl

^h^Others include *C. guilliermondii* (3)*, C. haemulonii* (2) and *C. pseudotropicalis* (1)

**Fig. 2:**
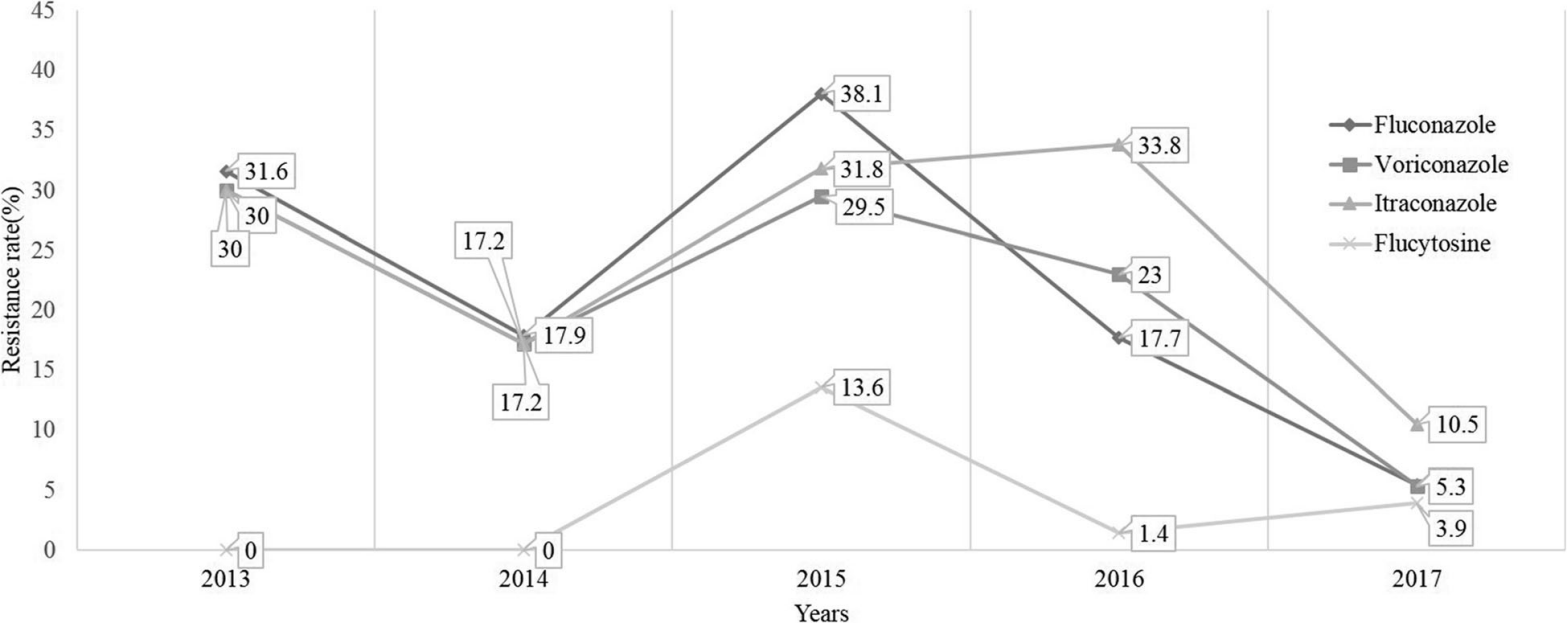
Resistance trend of four antifungal agents in *Candida* isolates from 2013 to 2017. All *Candida* strains (100%) were susceptible to amphotericin B, the resistance trend of which was not listed

The results of in vitro susceptibility testing of the *Candida* strain isolates are summarized in Table 2. Among the *Candida* strains, AMB demonstrated excellent in vitro activity against all *Candida* species. All *Candida* strains (100%) were susceptible to AMB, while 95.9% of the isolates were susceptible to 5-FC. The resistance rate of ITR was the highest in the *Candida* species (23.1%). A total of 18.6% of the isolates were resistant to FCA. VRC resistance was observed in 18.5% of all the *Candida* isolates evaluated. The resistance rate of *C. tropicalis* was the highest among the *Candida* species; most of the isolates were resistant to FCA (29.7%), ITR (40.5%) and VRC (27.0%). The percentages of yeast isolates with resistance and decreased susceptibility to FCA, ITR and VRC were significantly decreased during the 5 years of observation. (Fig. 2).

**Table 2:** In vitro antifungal susceptibility testing of 243 clinical isolates into 5 antifungal agents

Species (No of isolates)	Antifungal agent	MIC (μg/mL)			% Resistant
		Range	50%	90%	
*Candida albicans* (110)	Amphotericin B	≤0.5 to 1	≤0.5	≤0.5	0^b^
	Flucytosine	0.5 to > 8	0.5	1	5.5^b^
	Fluconazole	≤0.125 to 32	0.25	0.5	20.0
	Itraconazole	≤0.062 to 4	≤0.062	0.5	28.2^b^
	Voriconazole	≤0.062 to > 8	≤0.062	0.125	23.6
*Candida glabrata* (73)	Amphotericin B	≤0.5 to 1	≤0.5	1	0^b^
	Flucytosine	≤0.062 to > 16	0.125	0.5	2.7^b^
	Fluconazole	0.25 to> 128	8	32	11.0
	Itraconazole	≤0.25 to > 8	2	4	6.8^b^
	Voriconazole	≤0.062 to > 8	0.25	0.5	6.8^b^
*Candida tropicalis* (37)	Amphotericin B	≤0.5 to 1	≤0.5	1	0^b^
	Flucytosine	≤0.062 to 4	0.125	0.125	5.4^b^
	Fluconazole	0.25 to 64	1	2	29.7
	Itraconazole	≤0.062 to 64	0.125	0.25	40.5^b^
	Voriconazole	≤0.062 to > 8	≤0.062	0.125	27.0
*Candida krusei* (12)	Amphotericin B	≤0.5 to 2	≤0.5	1	0^b^
	Flucytosine	2 to 8	4	8	0
	Fluconazole^a^	–	–	–	–
	Itraconazole	≤0.062 to 4	0.125	0.5	33.4^b^
	Voriconazole	≤0.062 to > 8	0.125	0.25	25.0
*Candida parapsilosis* (5)	Amphotericin B	≤0.5 to 1	0.5	1	0
	Flucytosine	≤0.062 to 0.5	0.125	0.25	0
	Fluconazole	0.25 to 16	0.5	2	20.0
	Itraconazole	≤0.062 to 1	≤0.062	0.125	20.0^b^
	Voriconazole	≤0.062 to 4	≤0.062	0.125	20.0
Others^c^ (6)	Amphotericin B	≤0.5 to 1	≤0.5	1	0^b^
	Flucytosine	≤0.062 to 1	0.25	1	0^b^
	Fluconazole	0.5 to 64	1	4	16.7^b^
	Itraconazole	≤0.062 to 0.125	≤0.062	0.125	0^b^
	Voriconazole	≤0.062 to 0.5	≤0.062	0.125	0^b^
All of isolates (243)	Amphotericin B	–	–	–	0
	Flucytosine	–	–	–	4.1
	Fluconazole^a^	–	–	–	18.6
	Itraconazole	–	–	–	23.1
	Voriconazole	–	–	–	18.5

*MIC* minimal inhibitory concentration

^a^Resistance rate was based on the intrinsic resistance of *C*. *krusei* and did not follow the actual MICs

^b^The breakpoints of *Candida* spp. according to the manufacturer’s instructions of ATB FUNGUS 3 system

^c^Others include *C. guilliermondii* (3), *C. haemulonii* (2) and *C. pseudotropicalis* (1)

The outcome data at day 30 were available for 243 episodes. The hospital mortality rate was 12.3%. The mortality rates among *C. albicans*, *C. glabrata*, *C. tropicalis* and *Candida haemulonii* infections were 18.2% (20 out of 110 patients), 8.2% (6 out of 73 patients), 8.1% (3 out of 37 patients) and 50.0% (1 out of 2 patients), respectively. Sixteen patients died within 7 days of obtaining a positive culture. Respiratory dysfunction (39.2%), intensive care in adults (28.5%), neutropenia (28.6%), severe autoimmune diseases (27.3%) and cardiovascular diseases (25.6%) were frequently associated with mortality during the study period.

The univariate predictors of poor outcomes due to IC are shown in Table 3. For patients with IC, the variables associated with 30-day mortality were as follows: respiratory dysfunction, pulmonary infection, cardiovascular disease, chronic/acute renal failure, mechanical ventilation, abdominal surgery, intensive care in adults, septic shock and IC due to *C. albicans*. The results of the multivariate analysis are listed in Table 4. Respiratory dysfunction [*odds ratio* (OR), 9.80; 95% confidence interval (CI), 3.24–29.63; *P* < 0.001] and IC due to *C. albicans* (OR, 3.35; 95% CI, 1.13–9.92; *P* = 0.029) were the independent predictors of 30-day mortality.

**Table 3:** Factors associated with 30-day mortality by univariate analysis in patients with IC

Variable	30-day outcome		*P*-value
	Survived (*n* = 213)	Died (*n* = 30)	
Age,years (range)	57.8(17–91)	62.7(29–81)	0.127
Gender (male:female)	124:89	15:15	0.394
Underlying comorbidities (*n*, %)			
Gastrointestinal perforation	39 (18.3)	9 (30.0)	0.132
Respiratory dysfunction	31 (14.6)	20 (66.7)	< 0.001
Pulmonary infection	63 (29.6)	18 (60.0)	0.001
Cardiovascular disease	58 (27.2)	20 (66.7)	< 0.001
Neurological diseases	47 (22.1)	11 (36.7)	0.079
Gastrointestinal pathology	56 (26.3)	7 (23.3)	0.729
Chronic/acute liver disease	36 (16.9)	9 (30.0)	0.084
Chronic/acute renal failure	74 (34.7)	16 (53.3)	0.048
Solid tumour	19 (8.9)	3 (10.0)	0.847
Haematological malignancy	5 (2.3)	1 (3.3)	0.991
Severe autoimmune diseases	8 (3.8)	3 (10.0)	0.124
Diabetes mellitus	79 (37.1)	14 (46.7)	0.312
Burns	5 (2.3)	1 (3.3)	0.745
HIV/AIDS	11 (5.2)	2 (6.7)	0.732
Severe trauma	13 (6.1)	2 (6.7)	0.904
Risk factors (*n*,%)			
Presence of CVC	104 (48.8)	14 (46.7)	0.825
Mechanical ventilation	67 (31.5)	15 (50.0)	0.044
Receipt of corticosteroids	58 (27.2)	12 (40.0)	0.148
Total parenteral nutrition	100 (46.9)	19 (63.3)	0.093
Chemotherapy	10 (4.7)	1 (3.3)	0.737
Abdominal surgery	67 (31.5)	2 (6.7)	0.005
In the ICU at diagnosis	30 (14.1)	12 (40.0)	< 0.001
Neutropenia	5 (2.3)	2 (6.7)	0.185
Concomitant bacterial infections	67 (31.5)	14 (46.7)	0.098
Septic shock	35 (16.4)	11 (36.7)	0.008
Dialysis	21 (9.9)	4 (13.3)	0.558
Prior exposure to broad-spectrum antibiotics	188 (88.3)	27 (90.0)	0.780
Treatment with antifungal agents	71 (33.3)	12 (40.0)	0.471
Species, *n* (%)			
*C*. *albicans*	90 (42.3)	20 (66.7)	0.012
*C*. *glabrata*	67 (31.5)	6 (20.0)	0.200
*C*. *tropicalis*	34 (16.0)	3 (10.0)	0.395

*ICU* intensive care unit, *CVC* central venous catheter

**Table 4:** Factors associated with 30-day mortality by multivariate analysis

Variable	Odds ratio	95% confidence interval	*P*-value
Respiratory dysfunction	9.80	3.24–29.63	< 0.001
Pulmonary infection	1.09	0.39–3.06	0.871
Cardiovascular disease	2.37	0.81–6.92	0.114
Chronic/acute liver disease	0.61	0.18–2.06	0.426
Chronic/acute renal failure	1.86	0.63–5.45	0.262
Neurological diseases	0.93	0.33–2.61	0.884
Total parenteral nutrition	1.66	0.49–5.59	0.414
Mechanical ventilation	0.71	0.19–2.68	0.617
Abdominal surgery	0.22	0.04–1.08	0.062
In the ICU at diagnosis	2.22	0.70–6.89	0.176
Concomitant bacterial infections	0.88	0.30–2.60	0.821
Septic shock	1.91	0.58–6.29	0.289
*C*. *albicans*	3.35	1.13–9.92	0.029

*ICU* intensive care unit

## Discussion

This was a single-center retrospective study in a regional tertiary teaching hospital in southwest China. The inpatients mainly came from Luzhou, Neijiang, Yibin, Zigong, Chishui, and Zhaotong cities, which are prefecture-level medium and small cities (urban resident population < 1000,000 persons) in western China [22]. The medium and small cities were defined in accordance with the new standard for city-size classification in China (http://www.gov.cn/zhengce/content/2014-11/20/content_9225.htm). The urban population proportion, gross domestic product, income of residents, educational resources, sports resources, medical resources and air pollution in medium and small cities are less than those in large cities [23–25]; all the data are illustrated in the Additional file 1: Table S1.

Our data showed that the incidence of invasive *Candida* infection has increased steadily from 0.16 to 0.66 cases per 1000 admissions, which is similar to a metropolitan-based report in Beijing (0.13 to 0.55 cases per 1000 admissions), over the past 5 years [3]. However, the annual incidence of IC in our study (0.41 cases per 1000 admissions) was lower than those in metropolitan cities in China, such as Shanghai [26] (9.49 cases per 1000 admissions), and Nanjing [27] (0.71 to 0.85 cases per 1000 admissions). The reason for these different incidences might be that the subjects in Shanghai were mainly patients in ICUs wards, whereas the subjects in Nanjing were mainly patients with candidemia only.

In the study, *C. albicans* was the predominant species (50%) in the ICU. These results are consistent with a report from a multicenter prospective observational study in China [12]. However, in medical wards, the proportion of *C. glabrata* was higher than that of *C. albicans*, which was only exhibited in patients with hematological malignancies in the USA [28]. Although non-*C. albicans* species were significantly predominant, *C. albicans* was the most common isolate, consistent with reports from most other areas in China [26, 29–31]. However, in Nanjing [27], *C. tropicalis* was the most common isolate (28.6%). A possible reason may be related to the type of infection (only candidemia). In our study, we also found that *C. glabrata* was the second most common *Candida* species, similar to a report from a hospital in China [30], and hospitals in Denmark [32], Germany [33] and the USA [34]. Besides, we found that invasive *Candida* infections occurred more frequently in males of advanced age (age > 65), which was similar with those findings in USA [35], Denmark [32], Pakistan [10], China (Beijing) [30] and China (Nanjing) [27], however, the actual reasons for this finding are not fully understood. In those patients aged 50 to 65 years old, *C. glabrata* was the most common *Candida* species; this result was different from a 133 sample-based study in China [27].

With regard to resistance, resistance to FCA, ITR and VRC were common in *C. albicans* and non*-C. albicans* species. Our studies showed that the ITR resistance rate was the highest, at 23.1% in all *Candida* species, followed by FCA (18.6%), VRC (18.5%) and 5-FC (4.1%). However, none of the isolates in this study were resistant to AMB, and the susceptibility results in our city were higher than those in northern Ireland (susceptibility, 99.1%) [36]. In China, the resistance rates vary significantly among different cities. As shown in Table 5, the total resistance rates of all *Candida* species in the study were higher than those in metropolitan cities, especially Beijing [37] and Chongqing [38], which were higher than those in the China Survey of Candidiasis (China-SCAN) study [12]. Possible reasons may be related to the irrational use of antifungal agents, in healthcare and veterinary settings and by the general public [39]. In contrast, the resistance rates in our study were lower than those in Nanjing [27]. Regarding other countries, the antifungal resistance rate in our study was higher than that reported in northern Ireland in 2007–2011 [36]. The resistance rate of azoles in *C. albicans* (< 5%) [40] worldwide was significantly lower than that in our study (> 20.0%). The results of the ARTEMIS DISK Global Antifungal Surveillance Study showed that FCA- and VRC-resistant isolates of *C. glabrata* (15.7 and 10.0% resistance, respectively) [29] were approximately 1.5 times more prevalent than those in our study (11.0 and 6.8% resistance rates, respectively). The resistance rate of ITR in our study (23.1%) was higher than that in the USA (21.1%) [34]. The proportion of VRC-resistant isolates in our study was similar to that observed in India (18.5% vs 18.7%) [41]. In addition, our data showed that the resistance rates of FCA, ITR and VRC were higher in 2013 than in 2014, but the resistance rate of isolates significantly increased in 2015. The percentages of yeast isolates with resistance and decreased susceptibility to FCA, ITR and VRC showed a significant decrease from 2015 to 2017 (Fig. 2). Moreover, the annual resistance rate of antifungal agents was associated with the usage frequency of antifungal agents (Fig. 2 and Table 6). Therefore, it is necessary to surveil the changes in the resistance rate of antifungal agents.

**Table 5:** The resistance rates of antifungal agents in different cities in China

Species	Antifungal agent	Resistant (%)				
		Beijing	Chongqing	Nanjing	China-SCAN study	Luzhou
*C.albicans*	FCA	6.6	2.4	25.8	9.6	20.0
	VRC	2.9	5.6	0	0	23.6
	ITR	4.9	6.4	6.4	16.0	28.2
	5-FC		2.4			5.5
	AMB		0		0	0
*C.glabrata*	FCA	10.5	13.7	90.9	4.0	11.0
	VRC		9.1	0	6.0	6.8
	ITR		45.5	90.9	4.0	6.8
	5-FC		3.0			2.7
	AMB		0		0	0
*C.tropicalis*	FCA	10.6	2.2	68.4	6.0	29.7
	VRC	10.6	0	8.7	0	27.0
	ITR		0	34.2	31.3	40.5
	5-FC		0			5.4
	AMB		0		0	0
*C.krusei*	FCA			–	–	–
	VRC			0	0	25.0
	ITR			66.7	0	33.4
	5-FC					0
	AMB				0	0
*C.parapsilosis*	FCA		2.4	42.3	19.3	20.0
	VRC		0	0	3.6	20.0
	ITR		2.4	3.8	39.8	20.0
	5-FC		0			0
	AMB		0		0	0
Total	FCA	7.5		53.9		18.6
	VRC	5.4		2.2		18.5
	ITR	4.9		30.5		23.1
	5-FC					4.1
	AMB					0

*FCA* fluconazole, *ITR* itraconazole, *AMB* amphotericin B, *VRC* voriconazole, *5-FC* flucytosine

**Table 6:** The frequencies of antifungal usage in patients from 2013 to 2017

Antifungal agent	Patients *n* (%)^c^					
	Total (*n* = 243)	2013 (*n* = 20)	2014 (*n* = 29)	2015 (*n* = 44)	2016 (*n* = 74)	2017 (*n* = 76)
Amphotericin B(i.v.)	49 (20.2)	6(30.0%)	5 (17.2)	13 (29.5)	17 (23.0)	8 (10.5)
Fluconazole^a^(i.v.)	122 (52.8)	11 (55.0%)	20 (69.0)	19 (43.2)	30 (40.5)	42 (55.3)
Voriconazole(i.v.)	84 (34.6)	8 (40.0)	10 (34.5)	15 (34.1)	27 (36.5)	24 (31.6)
other^b^	20(13.3)	0	0	0	15(20.3)	5(6.5)

*i.v.* intravenous

^*a*^*C. krusei* is excluded

^*b*^other including caspofungin (intravenous) and itraconazole (oral)

^c^Some patients used more than two kind of antifungal drugs during the course of treatment

In this study, we analyzed the prognostic factors in patients with IC. Respiratory dysfunction, pulmonary infection, cardiovascular disease, chronic/acute renal failure, mechanical ventilation, abdominal surgery, intensive care in adults, septic shock and IC due to *C. albicans* were the predictors of mortality in the univariate analysis (*P* < 0.05). The number of prognostic factors (9 factors) in our city was clearly more than that in other regions of China, such as Beijing [3], Shanghai [26], Chongqing [38], Nanjing [27] and Jinan [4], and all the data are illustrated in the Additional file 2: Table S2. The differences in the prognostic factors in different cities might be related to the underlying diseases of patients in different cities, but the actual reasons are still unclear. Out of all the variables that were significantly associated with mortality in the univariate analysis, respiratory dysfunction and IC due to *C. albicans* were the predictive factors of mortality in the multivariate analysis. The results of some factors in this study agreed with the findings of a multicenter prospective observational study in China [31] and a retrospective analysis in Mexico [9]. In a study of critically ill patients, IC-associated mortality accounted for 58.6% [8]; the crude 30-day mortality rate in our study was only 12.3% in all patients with IC, which was substantially lower than the mortality rates reported in Italy (42.8%) [42], Mexico (38%) [9], Pakistan (52.1%) [10], Spain (30.0%) [43], Jinan (23.1%,China) [4] and Nanjing (26.0%,China) [27]. The reason for the high mortality rate might be that the research subjects in those countries and cities were mainly ICU-patients or patients with candidaemia. Those cases often had more severe underlying diseases and conditions, which are associated with high mortality, therefore having a higher risk of mortality [44, 45]. Conversely, the percentages of ICU patients and patient with candidemia in our study were only accounted for 17.3 and 47.7%, respectively. The lower mortality rate in our study was also identified in the Pu, S, et al. [38] with similar subjects in Chongqing. In our study, the mortality rate due to *C*. *haemulonii* was the highest (50%, 1/2), possibly because there were only two cases. The fatal case in our study was a critically ill patient in the ICU with respiratory dysfunction and severe underlying comorbidities. Excluding *C*. *haemulonii*, *C. albicans* was associated with the highest mortality rate (18.2%). In our study, respiratory dysfunction was the major cause of mortality (39.2%) in patients (OR, 9.80; 95% CI, 3.24–29.63; *P* < 0.001); Meanwhile, another study also indicated that hematological malignancy was also an independent predictor for 66.7% of death of in-hospital patients [27]. Certainly, the severity of the underlying comorbidities (hematological malignancy and respiratory dysfunction) considerably influenced the crude mortality rate in this patient population.

This study has several potential limitations. First, the subjects in our study were restricted to adult patients (> 16 years old). Therefore, our conclusions may not be extrapolated to pediatric patients. Second, due to the technical limitations of the clinical microbiology laboratory and the impact of hospital policies, there are no data on echinocandins in our hospital. Finally, this was a single-center retrospective study. Our data might be influenced by the distribution of the regional population, the level of medical intervention, and the distribution of patient types.

## Conclusion

This study shows that the incidence and mortality of IC in medium and small cities are lower than those in large cities, that the resistance rate to azoles is higher in medium and small cities than in large cities, and that the species distribution and risk factors in medium and small cities are different from those in large cities in China. This study provides reference data for future epidemiological and susceptibility studies on both antifungal agents and mortality due to IC in our hospital and in other hospitals in medium and small cities.

## Supplementary information

**Additional file 1: Table S1.** The different of urban population proportion, GDP, income, education resources, sports resources, medical resources and air population between Luzhou city and other cities in 2013

**Additional file 2: Table S2.** The difference of prognostic factors in difference studies about invasive candidiasis

## Data Availability

The datasets used and/or analysed during the current study are available from the corresponding author on reasonable request.

## Abbreviations

5-FC: flucytosine
AMB: Amphotericin B
CI: Confidence interval
CVC: Central venous catheter
FCA: Fluconazole
IC: Invasive candidiasis
ICU: Intensive care unit
IFD: Invasive fungal disease
ITR: Itraconazole
MALDI-TOF MS: Matrix-assisted laser desorption/ionization-time of flight mass spectroscopy
MIC: Minimal inhibitory concentration
OR: Odds ratio
VRC: Voriconazole
