# Supplementary material for: Surveillance study of the prevalence, species distribution, antifungal susceptibility, risk factors and mortality of invasive candidiasis in a tertiary teaching hospital in Southwest China
Source: BMC Infect Dis. 2019 Nov 7;19:939. doi: 10.1186/s12879-019-4588-9 (PMC6836498; doi:10.1186/s12879-019-4588-9)
Supplement: Supplementary file 1 — Additional file 1: Table S1. The different of urban population proportion, GDP, income, education resources, sports resources, medical resources and air population between Luzhou city and other cities in 2013 [file 12879_2019_4588_MOESM1_ESM.doc]

|  | Indicators | Luzhou^2^ | Jinan^3^ | Nanjing^4^ | Chongqing^5^ | Beijing^6^ | Shanghai^7^ |
| --- | --- | --- | --- | --- | --- | --- | --- |
| urban population^1^ | urban population（person） | <1 million | 1 million to 5 million | 5 million to 10 million | 5 million to 10 million | >10 million | >10 million |
| city-size* |  | medium-sized city | big city | metropolitan city | metropolitan city | mega city | mega city |
| urban population | urban population proportion (%) | 43.3% | 66.0% | 68.4% | 58.3% | 86.0% | 90.0% |
| gross domestic product | total GDP（100 million yuan） | 1104.5 | 5230.2 | 8011.8 | 12656.7 | 19500.6 | 21602.2 |
|  | per capita gross domestic product（yuan) | 26848 | 74993 | 98011 | 42795 | 93213 | 90092 |
| income(yuan） | rural average gross income per capita | 8455 | 13247 | 19446 | 10719 | 20418 | 20742 |
|  | urban average gross income per capita | 22821 | 35647 | 44226 | 26850 | 40321 | 43851 |
| education resources | primary schools | 270 | 602 | 339 | 4728 | 1093 | 759 |
|  | secondary schools | 239 | 299 | 276 | 1200 | 757 | 865 |
|  | regular institutions of higher education | 5 | 69 | 61 | 63 | 89 | 68 |
| sports resources | sports expenditures(10000yuan) | 7115 | 19262.7^10^ | 9683 | 91167 | 78522^12^ | 162670.9^11^ |
| medical resources | health care institutions(unit) | 4633 | 5369^13^ | 2315 | 18923^15^ | 10141^16^ | 4929^14^ |
|  | hospital | 101 | 197^13^ | 186 | 531^15^ | 647^16^ | 328^14^ |
|  | tertiary care hospital | 4 | 22^13^ | 25 | 28^17^ | 80^16^ | 38^14^ |
|  | doctors per 10000 persons(person) | 16 | 37^13^ | 25 | 16^15^ | 41^16^ | 41^14^ |
|  | hospital beds per 10000 persons(bed) | 43 | 68^13^ | 51 | 44^15^ | 55^16^ | 80^14^ |
| air pollution | quantity of days with good ambient air quality (day) | 298 | 61^9^ | 202 | 206 | 176^8^ | 241 |

**Additional file: Table S1**

The different of urban population proportion，GDP，income，education resources,sports resources,medical resources and air population between Luzhou city and other cities in 2013

**Note：**

**1. Due to the huge amount of data in 2013-2018, we only use the 2013 year’s data as an example to compare the differences of cities in the supplementary material.**

1. **The data unlabeled the reference which came from the city's statistical yearbook.**
2. **All data comes from the government information.**

*****: The classification of cities is only based on the new standard for city-size classification in China (the urban population of over 10 million is a mega city, 5 million to 10 million is a metropolitan city, and 1 million to 5 million is a big city, 500,000 to 1 million for medium-sized cities, and less than 500,000 for small cities)（<http://www.gov.cn/zhengce/content/2014-11/20/content_9225.htm>）

**Reference**

1. National Bureau of Statistics Of the People's Republic of China，the 6^th^ Population Census of china，http://www.stats.gov.cn/tjsj/pcsj/rkpc/6rp/indexch.htm.
2. Statistics Bureau of luzhou , Luzhou statistical yearbook 2014, [http://tjj.luzhou.gov.cn/tjsj/tjnj/lztjjoldtjnj2014/lztjj7567/lztjj7568.](http://tjj.luzhou.gov.cn/tjsj/tjnj/lztjjoldtjnj2014/lztjj7567/lztjj7568泸州市情况)
3. Statistics Bureau of Jinan , Jinan statistical yearbook 2014, <http://jntj.jinan.gov.cn/art/2018/7/13/art_27523_2474569.html>.
4. Nanjing Municipal Bureau of Statistics, Nanjing statistical yearbook 2014, <http://221.226.86.104/file/2014/index.htm>.
5. Chongqing Municipal Bureau of Statistics, Chongqing statistical yearbook 2014, http://tjj.cq.gov.cn//tjnj/2014/indexch.htm.
6. Beijing Municipal Bureau of Statistics, Beijing Statistical yearbook 2014, http://tjj.beijing.gov.cn/nj/main/2014_tjnj/index.htm.
7. Shanghai Municipal Bureau of Statistics, Shanghai Statistical yearbook 2014, <http://www.stats-sh.gov.cn/html/sjfb/201701/1000200.html>.
8. Beijing Municipal Ecological Environment Bureau, The average annual concentration of PM 2.5 in Beijing in 2013 was 89.5 μg/m^3^ , <http://sthjj.beijing.gov.cn/bjhrb/xxgk/jgzn/jgsz/jjgjgszjzz/xcjyc/xwfb/607251/index.html>.
9. Ecological Environment Bureau of Jinan, 2013 Jinan environmental quality briefing, <http://jnepb.jinan.gov.cn/art/2014/9/17/art_10451_1113023.html>
10. Sports Bureau of Jinan, 2013 Jinan city sports bureau department final accounts, <http://jnstyj.jinan.gov.cn/module/download/downfile.jsp?classid=-1&filename=1805021110377883762.pdf>.
11. Shanghai Administration of Sports, 2013 annual department final account of Shanghai administration of sports, <http://tyj.sh.gov.cn/General/CzxxDetail/3e7f1ab7-3e58-47b3-8749-f438345a5cf7>.
12. Beijing Municipal Bureau of Sports, 2013 departmental budget revenue and expenditure budget summary , <http://tyj.beijing.gov.cn/bjsports/zfxxgk_/czyjs/1220296/index.html>.
13. Jinan Municipal Health Commission, Statistical report on the development of health care in Jinan city in 2013 , <http://jnmhc.jinan.gov.cn/art/2014/4/12/art_25167_2599053.html?xxgkhide=1>.
14. Shanghai Municipal Health Commission, Health data of shanghai in 2013, [http://wsjkw.sh.gov.cn/tjsj2/20180815/58452.html.](http://wsjkw.sh.gov.cn/tjsj2/20180815/58452.html上海市医疗卫生情况)
15. Chongqing Municipal Health Commission, Chongqing municipal health commission statistical yearbook 2015, <http://wsjkw.cq.gov.cn/res/pdfjs/web/viewer.html?file=L3UvY21zL3d3dy8yMDE5MDIvMTkxNTAzNDJxZ2s0LnBkZg==&title=JUU5JTg3JThEJUU1JUJBJTg2JUU1JThEJUFCJUU3JTk0JTlGJUU1JTkyJThDJUU4JUFFJUExJUU1JTg4JTkyJUU3JTk0JTlGJUU4JTgyJUIyJUU3JUJCJTlGJUU4JUFFJUExJUU1JUI5JUI0JUU5JTg5JUI0JUVGJUJDJTg4JUU3JUJCJTg4JUVGJUJDJTg5LnBkZg==>.
16. Beijing Municipal Health Commission information center, Overview of Beijing's health work in 2013, <http://www.phic.org.cn/sznj/bjwsjsgzgk/201812/t20181204_258783.html.>
17. Chongqing Municipal Health Commission, The main data of health in recent years in Chongqing city, <http://wsjkw.cq.gov.cn/res/pdfjs/web/viewer.html?file=L3UvY21zL3d3dy8yMDE5MDIvMTkxNTAwMjM3emQxLnBkZg==&title=MjAxNSVFNSVCOSVCNCVFNCVCOCVCQiVFOCVBNiU4MSVFNiU5NSVCMCVFNiU4RCVBRSVFNSU4NiU4NSVFNSVBRSVCOS5wZGY=.>
