# Supplementary material for: Surveillance study of the prevalence, species distribution, antifungal susceptibility, risk factors and mortality of invasive candidiasis in a tertiary teaching hospital in Southwest China
Source: BMC Infect Dis. 2019 Nov 7;19:939. doi: 10.1186/s12879-019-4588-9 (PMC6836498; doi:10.1186/s12879-019-4588-9)
Supplement: Supplementary file 2 — Additional file 2: Table S2. The difference of prognostic factors in difference studies about invasive candidiasis [file 12879_2019_4588_MOESM2_ESM.doc]

**Additional file 2: Table S2**

| The difference of prognostic factors in difference studies about invasive candidiasis | | |
| --- | --- | --- |
| Reference | prognostic factors | |
|  | same or similar | different |
| This study | 1. mechanical ventilation^a^, 2. abdominal surgery^b^, 3. septic shock^c^, 4. IC due to *C*. *albicans*^d^, | 1. Respiratory dysfunction, 2. pulmonary infection, 3. cardiovascular disease, 4. chronic/acute renal failure, 5. intensive care in adults, |
| 26 | 1. invasive mechanical ventilation^a^ | 1. total parenteral nutrition, 2. prior fluconazole therapy, 3. patient age over 65 years |
| 27 | 1. bacterial sepsis^c^, 2. tracheal intubation^a^, | 1. head trauma, 2. increased peripheral WBC count |
| 28 | 1. complicated abdominal surgery^b^, 2. candidemia due to *C. tropicalis*^d^*，* | 1. presence of central venous catheter, 2. poor treatment with fluconazole 3. neutropenia, |
| 45 | 1. receipt of mechanical ventilation^a^， | 1. APACHE II score ≥20, 2. absence of antifungal therapies |
| 3 |  | IC patients with *C. albicans:*   1. solid tumor, 2. Hypoproteinemia, 3. older age |
|  |  | IC patients with *C. non-albicans spp*:   1. hospital length of stay, 2. usage of corticosteroids, 3. duration on corticosteroids, 4. Chemotherapy, 5. hematologic malignancy, 6. usage of glycopeptides, 7. neutropenia |

a, b ,c and d: Same superscript means the prognostic factors are the same or similar.

**Reference**

3. Pu S, Niu S, Zhang C, Xu X, Qin M, Huang S, Zhang L: Epidemiology, antifungal susceptibilities, and risk factors for invasive candidiasis from 2011 to 2013 in a teaching hospital in southwest China. J Microbiol Immunol Infect 2017, 50(1):97-103.

1. Wang L, Tong Z, Wang Z, Xu L, Wu Y, Liu Y, Wu L: Single-center retrospective study of the incidence of, and risk factors for, non-C. albicans invasive candidiasis in hospitalized patients in China. Med Mycol 2014, **52**(2):115-22.

27. Wu JQ, Zhu LP, Ou XT, Xu B, Hu XP, Wang X, Weng XH: Epidemiology and risk factors for non-Candida albicans candidemia in non-neutropenic patients at a Chinese teaching hospital. *Med Mycol* 2011, 49(5):552-5.

28. Ma CF, Li FQ, Shi LN, Hu YA, Wang Y, Huang M, Kong QQ: Surveillance study of species distribution, antifungal susceptibility and mortality of nosocomial candidemia in a tertiary care hospital in China. *BMC Infect Dis* 2013, 13:337.

45. Wang H, Liu N, Yin M, Han H, Yue J, Zhang F, Shan T, Guo H, Wu D: The epidemiology, antifungal use and risk factors of death in elderly patients with candidemia: a multicentre retrospective study. BMC Infect Dis 2014, 14:609.
